# c-KIT inhibitors reduce pathology and improve behavior in the Tg(SwDI) model of Alzheimer’s disease

**DOI:** 10.26508/lsa.202402625

**Published:** 2024-07-15

**Authors:** Max Stevenson, Michaeline L Hebron, Xiaoguang Liu, Kaluvu Balaraman, Christian Wolf, Charbel Moussa

**Affiliations:** 1 Translational Neurotherapeutics Program, Laboratory for Dementia and Parkinsonism, Department of Neurology, Georgetown University Medical Center, Washington DC, USA; 2 Medicinal Chemistry Shared Resource, Department of Chemistry, Georgetown University Medical Center, Washington DC, USA

## Abstract

Treatments for Alzheimer’s disease have primarily focused on removing brain amyloid plaques to improve cognitive outcomes in patients. We developed small compounds, known as BK40143 and BK40197, and we hypothesize that these drugs alleviate microglial-mediated neuroinflammation and induce autophagic clearance of neurotoxic proteins to improve behavior in models of neurodegeneration. Specificity binding assays of BK40143 and BK40197 showed primary binding to c-KIT/Platelet Derived Growth Factor Receptors (PDGFR)α/β, whereas BK40197 also differentially binds to FYVE finger-containing phosphoinositide kinase (PIKFYVE). Both compounds penetrate the CNS, and treatment with these drugs inhibited the maturation of peripheral mast cells in transgenic mice, correlating with cognitive improvements on measures of memory and anxiety. In the brain, microglial activation was profoundly attenuated and amyloid-beta and tau were reduced via autophagy. Multi-kinase inhibition, including c-KIT, exerts multifunctional effects to reduce neurodegenerative pathology via autophagy and microglial activity and may represent a potential therapeutic option for neurodegeneration.

## Abstract (precis)

Brain-penetrant c-KIT inhibitors, BK40143 and BK4019,7 regulate harmonious mechanisms via autophagy and regulation of mast-cell-microglial communication and improve cognition in Alzheimer’s models.

## Graphical Abstract

**Figure:**
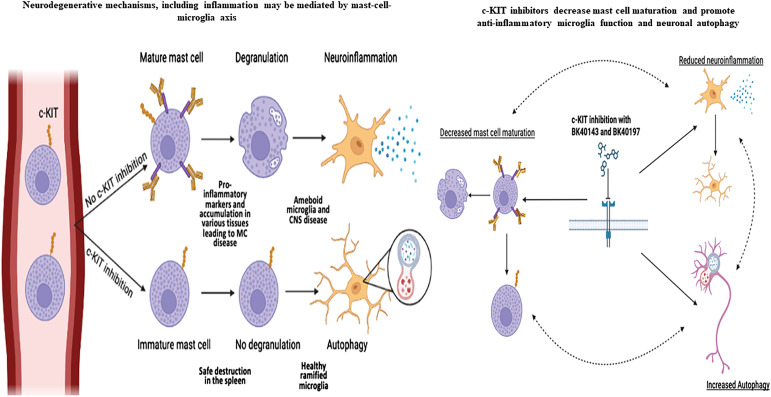

## Introduction

Alzheimer’s disease (AD) is a neurodegenerative disorder of aging that currently affects at least 55 million people worldwide (Long et al, 2023). Primary symptoms of AD include impairments in cognitive function and loss of memory as well as psychological symptoms such as depression and anxiety. The most well-known pathological features of AD are the accumulation of amyloid-beta (Aβ) plaques composed of a cleaved fragment of amyloid precursor protein (APP) and hyperphosphorylated tau (pTau) tangles, which are believed to synergistically contribute to synaptic dysfunction, neuron death, and cognitive decline (Mattson, 2004; Soria-Lopezet al, 2019). Another feature of AD that contributes to neurodegeneration is the prevalence of neuroinflammation, which involves activation of microglia, immune cells responsible for removing extracellular waste and releasing pro-inflammatory cytokines and chemokines to promote the immune response (Lyman et al, 2014; Subhramanyam et al, 2019). Indeed, neuroinflammation is hypothesized to precede or trigger neurotoxic protein aggregation (Kempuraj et al, 2016; Kaur et al, 2019). Disease-modifying therapies are being developed aimed at alleviating progression of dementia via removal of brain amyloid but approved anti-amyloid and other drug therapies have demonstrated only modest, temporary, and palliative benefits (Maia & Sousa, 2019; Cummings et al, 2020). As such, continuing to pursue novel therapies aimed at alleviating these various factors associated with AD pathogenesis is of paramount importance.

One class of enzyme being explored in both preclinical and clinical settings for efficacy in treating neurodegenerative diseases is tyrosine kinases (TKs) (Hebron et al, 2017; Fowler et al, 2019; Jiang & Ji, 2019). Many of these enzymes, which can exist as either membrane-bound or cytoplasmic, are upregulated in postmortem brains from AD and Parkinson’s Disease (PD) patients (Hebron et al, 2017; Fowler et al, 2019), although the distinct roles that they play in neurodegenerative disease remain relatively unexplored. Recent data have implicated TK activity in AD pathology, as several downstream substrates such as PI3K, MEK, and JAK are associated with cytokine production and autophagic clearance of cell waste (Lemmon & Schlessinger, 2010; Fraser et al, 2017; Hebron et al, 2017). Pharmacological inhibition of TKs, such as Abelson (Abl), Discoidin Domain Receptor-1 (DDR1), Platelet Derived Growth Factors (PDGFRα/β) and fused in sarcoma (Src), diminishes AD pathology in APP transgenic mice, suggesting this as a potential therapeutic strategy (Hebron et al, 2017; Fowler et al, 2019). In addition, nilotinib, an Abl/DDR inhibitor, has been shown by to reduce pathological markers in AD and PD patients (Hebron et al, 2013a; Lonskaya et al, 2015; Turner et al, 2020; Fowler et al, 2021; Stevenson et al, 2023), and improve clinical outcomes in PD (Pagan et al, 2019, 2020, 2021). Bosutinib, an Abl/Src inhibitor, reduces pathological markers and improves activities of daily living in patients diagnosed with Dementia with Lewy Bodies (DLB) (Hebron et al, 2013b, 2014; Mahul-Mellier et al, 2014; Lonskaya et al, 2015; Javidnia et al, 2017; Pagan et al, 2022). Masitinib, a c-KIT inhibitor, is currently in late-stage clinical trials for AD (Folch et al, 2015) and multiple sclerosis (Arsenault et al, 2022), but displays poor brain penetrance. Collectively, these data suggest that multi-kinase inhibition provides an effective means of alleviating neurodegenerative disease pathologies (Hebron et al, 2013b, 2014; Mahul-Mellier et al, 2014; Lonskaya et al, 2015; Javidnia et al, 2017; Fowler et al, 2019). We synthesized two novel small molecules known as BK40143 and BK40197 (Fowler et al, 2020) and hypothesized that treatment with these compounds will mitigate AD pathology via autophagic clearance of toxic proteins (i.e., Aβ, ptau, and α-synuclein) and alleviation of neuroinflammation to improve behavioral outcomes in models of neurodegeneration.

## Results

### BK40143 and BK40197 are selective for specific tyrosine kinases

**Figure 1.**
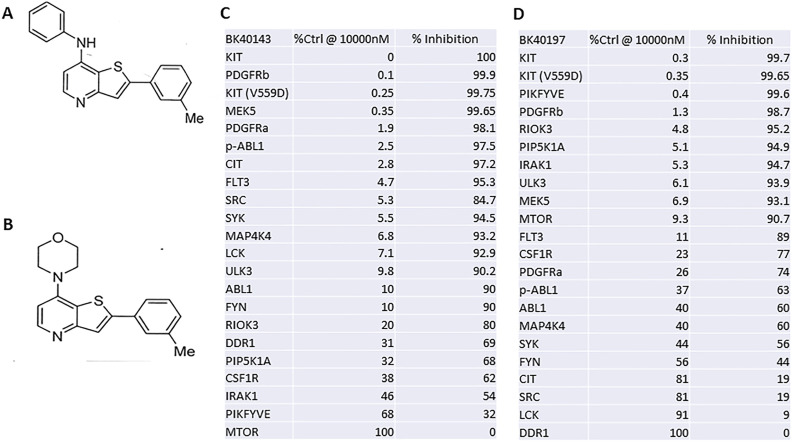
BK40143 and BK40197, two novel c-KIT inhibitors. **(A, B, C, D)** Chemical structures of (A) BK40143 and (B) BK40197, two novel inhibitors of c-KIT, with corresponding competitive binding assay values for select kinases ((C) BK40143, (D) BK40197). Source data are available for this figure.

We performed screening analysis via KinomeScan, a competitive binding assay, to determine the selectivity profiles of BK40143 (Fig 1A) and BK40197 (Fig 1B) against over 450 distinct kinases (Fig 1C and D). Primary screening results and corresponding compound/target affinities indicated POC (percentage of control) values with tighter binding (higher affinity) interactions associated with lower POC values and weaker binding (lower affinity) associated with higher POC values. We found that the lowest POC range of 0 ≤ x < 0.1 corresponded with BK40143 and BK40197 (Fig 1C and D) completely abolishing ligand binding of human c-KIT at 0 and 0.3 POC, respectively. The gain-of-function c-KIT (V559D) mutation (Hirota et al, 1998) that is associated with c-KIT activation resulting in aberrant mast cell growth and accumulation of mast cells in tissues (El-Agamy, 2012), showed similar binding affinity of BK40143 (0.25 POC) and BK40197 (0.35 POC). BK40143 also prevented ligand binding against PDGFRβ (0.1 POC), whereas BK40197 showed lower affinity with a POC 1.3. Interestingly, BK40197 displayed 0.4 POC against the autophagy-related protein complex, FYVE finger-containing phosphoinositide kinase (PIKFYVE) (Rivero-Rios & Weisman, 2022). BK40143 also displayed a POC range of 1 ≤ x < 10 against several other targets, including PDGFRα, phosphorylated Abl, p-Abl (2.5), the serine/threonine Citron Rho kinase, CIT (2.8), the FMS-like tyrosine kinase-3, FLT3 (4.7), Src (5.3), and SRC family tyrosine kinases that activate T-cell signaling, including spleen tyrosine kinase, SYK (5.5), mitogen-activated protein (MAP)4K4 (6.8), lymphocyte-specific protein tyrosine kinase, LCK (7.1), and Unc-51-like autophagy kinase, ULK3 (9.8), whereas BK40197 displayed affinity to a plethora of other autophagy and inflammation related kinases, including the anti-inflammatory serine/threonine-protein kinase RIO3 (RIOK3) (4.8), Phosphatidylinositol-4-phosphate 5-kinase type-1 alpha (PIP5K1A) (5.1), interleukin-1 receptor-associated kinase 1(IRAK1) (5.3), ULK3 (6.1), the threonine/tyrosine mitogen-activated protein kinase kinase (MEK5) (6.9), and the mammalian Target of Rapamycin, mTOR (9.3).

### BK40143 and BK40197 induce autophagic clearance of α-synuclein in vitro

**Figure 2.**
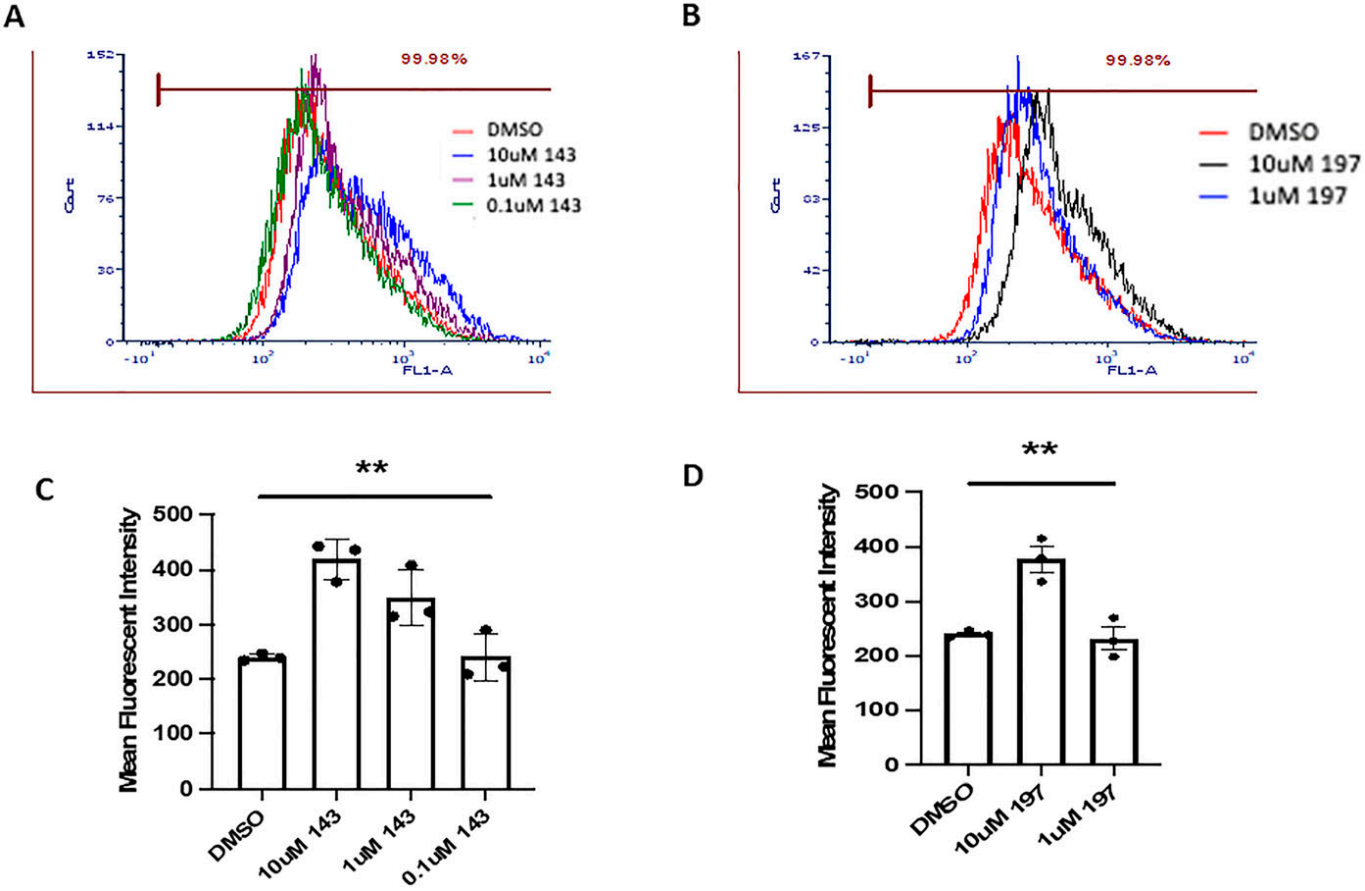
In vitro target engagement analysis of BK40143 and BK40197. **(A, B, C, D)** Flow cytometric analysis of SH-SY5Y cells transduced with α-synuclein revealed increased production of autophagic vacuoles in cells treated with (A, B) BK40143 (*P* = 0.001 two-tailed *t* test) and (C, D) BK40197 (*P* = 0.004, two-tailed *t* test). Source data are available for this figure.

Binding specificity of BK40143 and BK40197 revealed binding to major protein kinases involved in autophagy, including ULK3 (Gonzalez-Rodriguez et al, 2022), mTOR (Wang & Zhang, 2019), PDGFRs (Zhang et al, 2021b), PIKFYVE (O’Connell & Vassilev, 2021), as well as c-KIT (Lee et al, 2013). To establish that treatment with BK40143 and BK40197 induces autophagy, human SH-SY5Y neuroblastoma cells were transfected with an α-synuclein plasmid before being treated with rapamycin/chloroquine as a positive control, DMSO as a negative control, or an ascending dose (10, 1, or 100 μM) of either BK40143 or BK40197 for 8 h. Cells were then harvested and treated using the Abcam Autophagy Assay kit, which uses a cationic amphiphilic tracer to stain autophagic vacuoles, before analysis via flow cytometry. We found that treatment with 10 μM of both BK40143 (Fig 2A and C) and BK40197 (Fig 2B and D) resulted in significant increases in labelled cells, indicating induction of autophagy in cells stressed with α-synuclein.

### BK40143 and BK40197 inhibit peripheral mast cell maturation in a mouse model of AD

**Figure 3.**
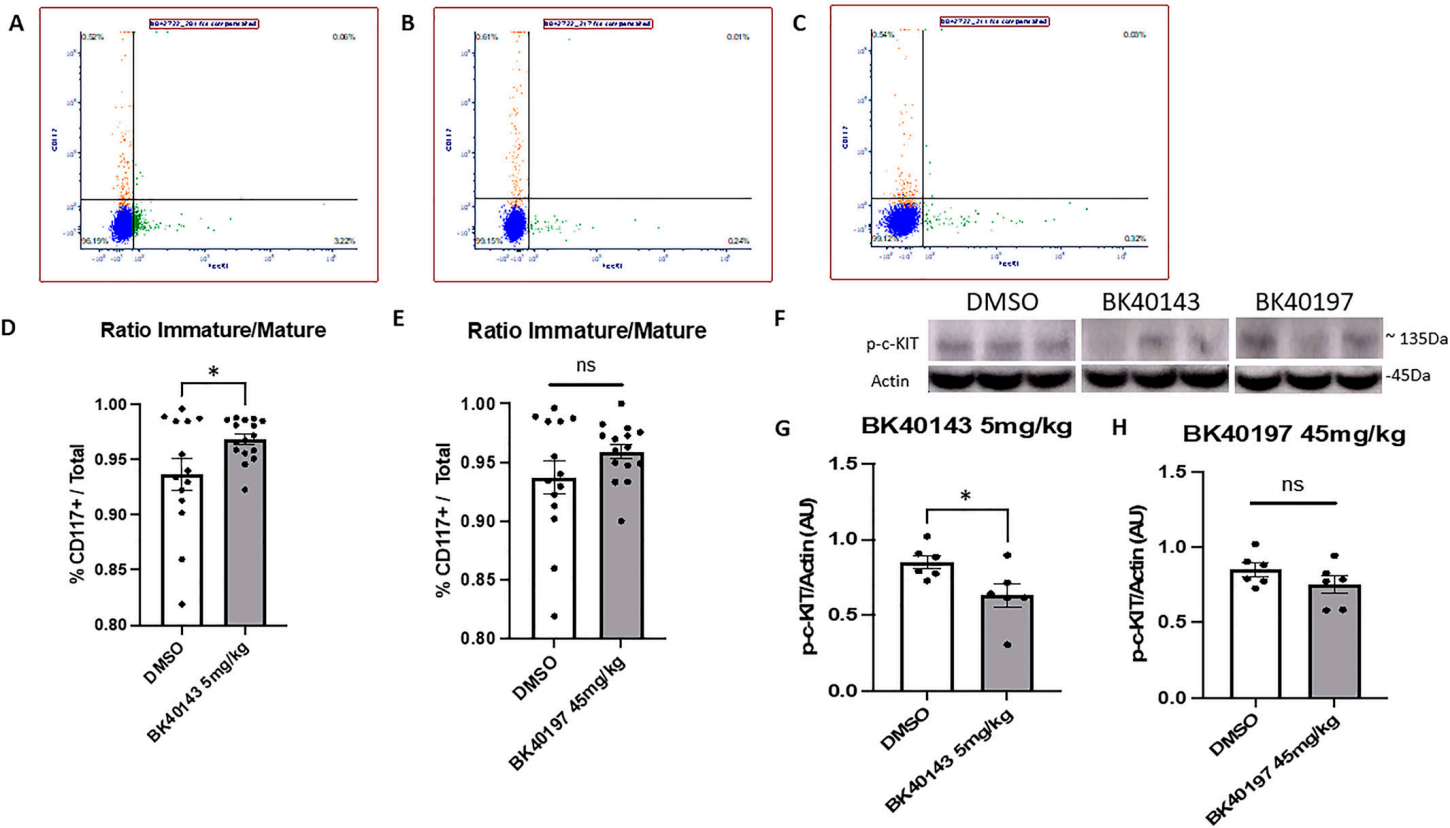
BK40143 and BK40197 inhibit mast cell proliferation in vivo. **(A, B, C, D, E)** Flow cytometric analysis using antibodies conjugated against c-KIT (CD117) and FcεR1 revealed that TgAPP mice injected with DMSO displayed (A) decreased ratios of immature versus mature mast cells compared with mice treated with (B, D) 5 mg/kg BK40143 (*P* = 0.03, two-tailed *t* test) and (C, E) 45 mg/kg BK40197 (*P* = 0.07, two-tailed *t* test). **(F, G, H)** Western blot revealed decreased levels of phospho-c-KIT in the brains of TgAPP mice treated with (G) 5 mg/kg BK40143 (*P* = 0.03, two-tailed *t* test) and (H) 45 mg/kg BK40197 (*P* = 0.19, two-tailed *t* test). Source data are available for this figure.

c-KIT is responsible for mast cell development and homeostasis (Lake et al, 1978; Linnekin, 1999; Kitamura et al, 2006). TKs are the central driving force for processes leading to mast cell activation (Parravicini et al, 2002; Gomez et al, 2005). Multiple receptors are capable of mediating or modifying mast cell activation (Kraft & Kinet, 2007; Kuehn & Gilfillan, 2007), the major receptor responsible being FcεRI, the high affinity receptor for immunoglobulin E (IgE) (Rivera & Gilfillan, 2006). Mast cells are tissue-resident cells of hematopoietic lineage which are derived from CD13^+^CD34^+^KIT(CD117)^+^ bone marrow pluripotent progenitors (Kirshenbaum et al, 1999). Although mast cells play an important role in the innate immune system (Marshall, 2004; Galli et al, 2005), these cells are also responsible for the detrimental exaggerated reactions to antigen (Galli et al, 2008) that follow the release of potent inflammatory mediators as a consequence of receptor-mediated mast cell activation (Metcalfe et al, 1997). The progression of these cells to fully mature mast cells is dependent on c-KIT activation which occurs because of stem cell factor-induced c-KIT dimerization and auto-phosphorylation (Kirshenbaum et al, 1999). To verify the functional effects of BK40143 and BK40197 on c-KIT inhibition, we investigated whether inhibition of c-KIT with BK40143 and BK40197 affects peripheral mast cell populations in vivo. We collected whole blood samples from Tg(SwDI) (referred to as TgAPP) mice treated with 5 or 45 mg/kg of each drug, respectively, or DMSO for 6 wk before isolating mast cells for analysis via flow cytometry (Fig 3A–C). Whole blood was sorted for mast cells using a triple gating strategy: 647 anti-CD45 to isolate leukocytes, PE anti-c-KIT to isolate mast cells, and APC/fire anti-FcεR1α to identify mature mast cells. The ratio of immature-to-mature mast cells (c-KIT+ versus c-KIT+/ FcεR1α+) was compared between c-KIT inhibited and DMSO-treated controls to determine the functional consequence of c-KIT inhibition on peripheral mast cell maturation. We found that mice treated with 5 mg/kg BK40143 (Fig 3B and D) exhibit a significantly greater ratio of immature-to-mature mast cells when compared with DMSO-treated mice (Fig 3A and D), indicating that c-KIT inhibition with BK40143 prevents maturation of peripheral mast cell progenitors. In mice treated with BK40197, we saw a similar, albeit not statistically significant (*P* = 0.07) increase in immature-to-mature mast cell ratio (Fig 3C and E), further signifying prevention of mast cell progenitor maturation. We further demonstrated c-KIT inhibition in the brains of our mice by performing Western blot for phospho-c-KIT on lysates from drug treated versus control mice. We observed a significant decrease in phospho-c-KIT levels in BK40143-treated mice (Fig 3F and G), whereas BK40197-treated mice displayed a similar, albeit not statistically significant, trend (Fig 3F and H). Collectively, the data confirm the screening experiment and indicates that treatment with BK40143 and BK40197 is sufficient to prevent mast cell proliferation in vivo, via c-KIT inhibition.

### Pharmacokinetics

**Figure 4.**
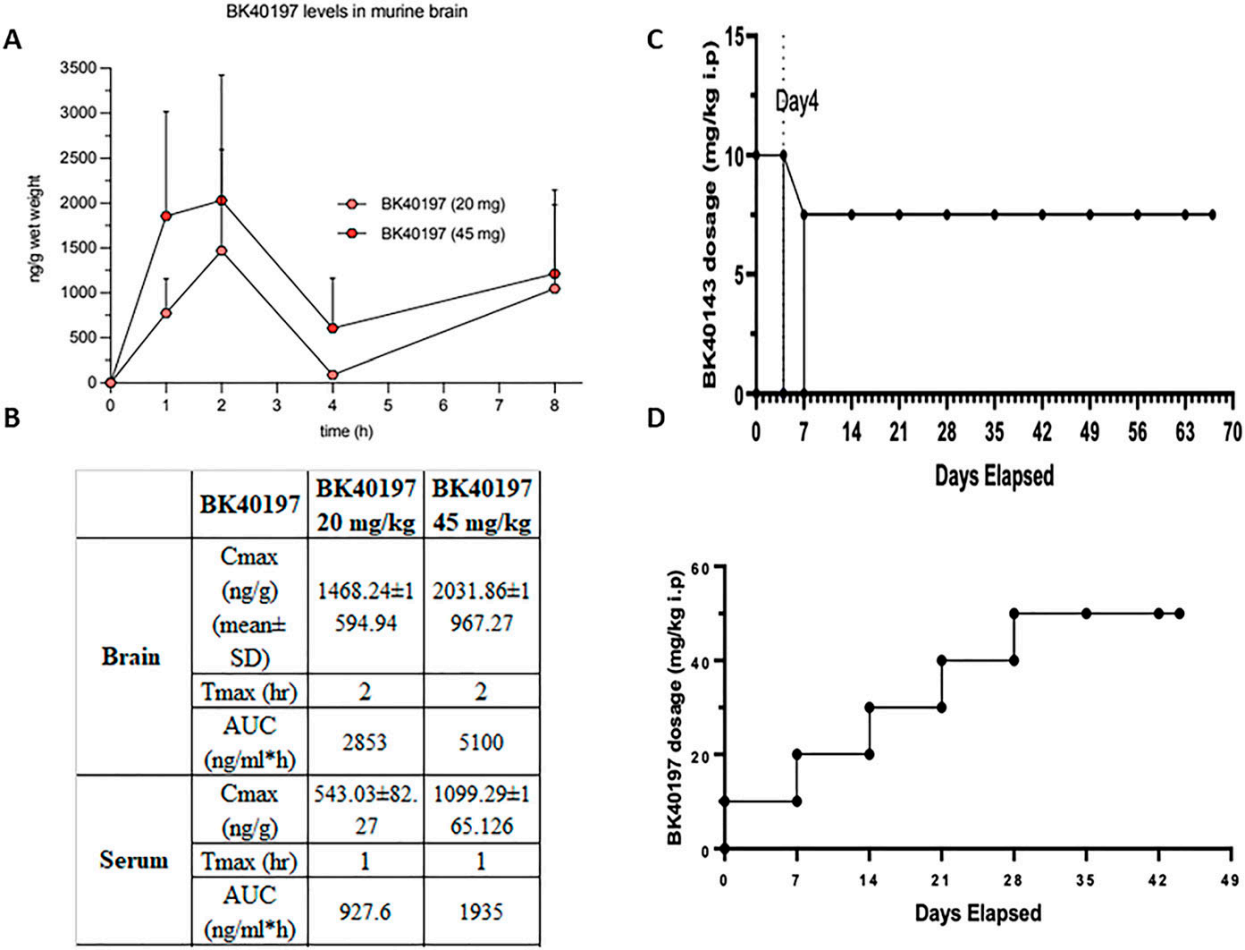
Pharmacokinetic properties of BK40197. **(A, B)** Pharmacokinetic analysis revealed (A, B) brain penetrance of BK40197 at multiple doses. **(C, D)** Administration of (C) BK40143 and (D) BK40197 in WT mice revealed the maximum tolerable dose for each drug in vivo. Source data are available for this figure.

We previously demonstrated that BK40143 was brain penetrant (Fowler et al, 2020). Pharmacokinetic (PK) analysis of BK40197 showed it to also be brain penetrant (Fig 4A and B), with a brain to serum ratio of 25.1% at 20 mg/kg and 20.1% at 45 mg/kg, indicating that both our compounds cross the blood-brain barrier (BBB). We then treated WT mice with ascending doses of BK40143 and BK40197 to determine the maximum tolerable dose for each drug in vivo. Multiple ascending dose treatments revealed the maximum tolerable dose of BK40143 to be 7.5 mg/kg injected IP (Fig 4C), whereas a similar paradigm revealed the maximum tolerable IP dose of BK40197 to be 45 mg/kg (Fig 4D). This identified our compounds as brain penetrant and tolerable in mice, allowing us to test them as potential treatments for neurodegenerative diseases.

### BK40143 and BK40197 improve behavior in mouse models of neurodegeneration

**Figure 5.**
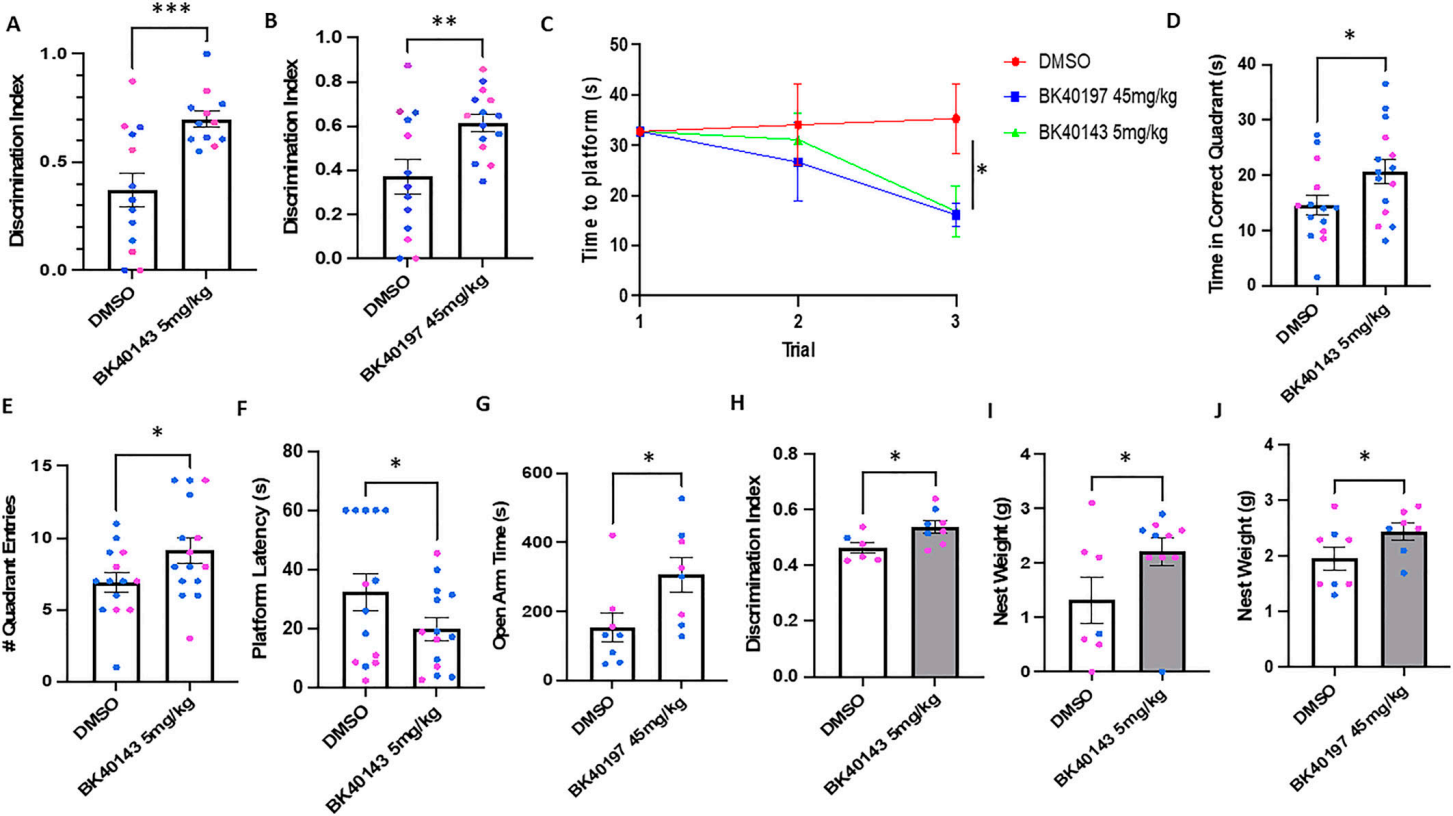
BK40143 and BK40197 improve behavioral outcomes in animal models of proteinopathies. **(A, B)** Male (blue) and female (red) 12-mo-old TgAPP mice tested via novel object recognition displayed significantly improved outcomes when treated with (A) 5 mg/kg BK40143 (*P* = 0.0003) or (B) 45 mg/kg BK40197 (*P* = 0.006) compared with DMSO-treated controls. **(C, D, E, F)** These mice also displayed improved recall during (C) Morris water maze training trials (*P* = 0.04), whereas mice treated with 5 mg/kg BK40143 showed similar improvements on (D, E, F) Morris water maze endpoint measures (time in correct quadrant: *P* = 0.02, quadrant entries: *P* = 0.03. platform latency: *P* = 0.05). **(G, H, I, J)** TgAPP mice treated with 45 mg/kg BK40197 (G) spend more time in the open arm of the elevated plus maze compared to DMSO-treated controls (*P* = 0.02). 12-mo-old A53T mice treated with 5 mg/kg BK40143 display improved outcomes on (H) novel object recognition (*P* = 0.03), while A53T mice treated with (I) 5 mg/kg BK40143 (*P* = 0.04) or (J) 45 mg/kg BK40197 (*P* = 0.05) showed significant improvements in nesting behavior. Source data are available for this figure.

To examine the behavioral outcomes following c-KIT inhibition with BK40143 and BK40197, we treated TgAPP mice with either 5 or 45 mg/kg IP, respectively, or DMSO for 6 wk, before performing behavioral analysis. We used the novel object recognition test, a memory assessment that indicates mild cognitive impairment, the Morris water maze, a measure of spatial memory that demarcates more severe memory impairments, the elevated plus maze, a measure of anxiety-like behavior, and nesting, a metric of normative mouse behavior. We discovered that mice treated with both BK40143 (Fig 5A) and BK40197 (Fig 5B) spent significantly greater amounts of time exploring the novel object than did DMSO-treated controls, indicating improved recall on the novel object recognition test. We also found that mice treated with both drugs were able to locate the platform significantly quicker on later training trials than the control mice (Fig 5C), indicating improved spatial memory after drug treatment. Interestingly, only mice treated with BK40143 showed improvement on measures of memory on the probe day of the water maze (time in the correct quadrant [Fig 5D], number of quadrant entries [Fig 5E], and latency to the platform [Fig 5F]), demonstrating that treatment with BK40143 correlates with greater improvement in spatial memory. Furthermore, we found that only TgAPP mice treated with BK40197 spent significantly greater time in the open arms of the elevated plus maze than the DMSO-treated controls (Fig 5G), indicating that treatment with BK40197 may be sufficient to alleviate cognitive symptoms associated with AD pathology, such as anxiety. Lastly, we examined memory and nesting behavior in A53T mice treated with our drugs and observed significant improvements on novel object recognition in mice treated with BK40143 (Fig 5H), as well as significant decreases in nest weight, indicative of decreased nestlet shredding behavior, in mice treated with both BK40143 (Fig 5I) and BK40197 (Fig 5J). Together, these data emphasize the potential of these two compounds in alleviating cognitive-behavioral deficits associated with AD and identifies c-KIT inhibition as a putative method for therapeutic intervention when treating neurodegenerative disorders.

### BK40143 and BK40197 reduce concentrations of amyloid-beta and pTau via autophagy

**Figure 6.**
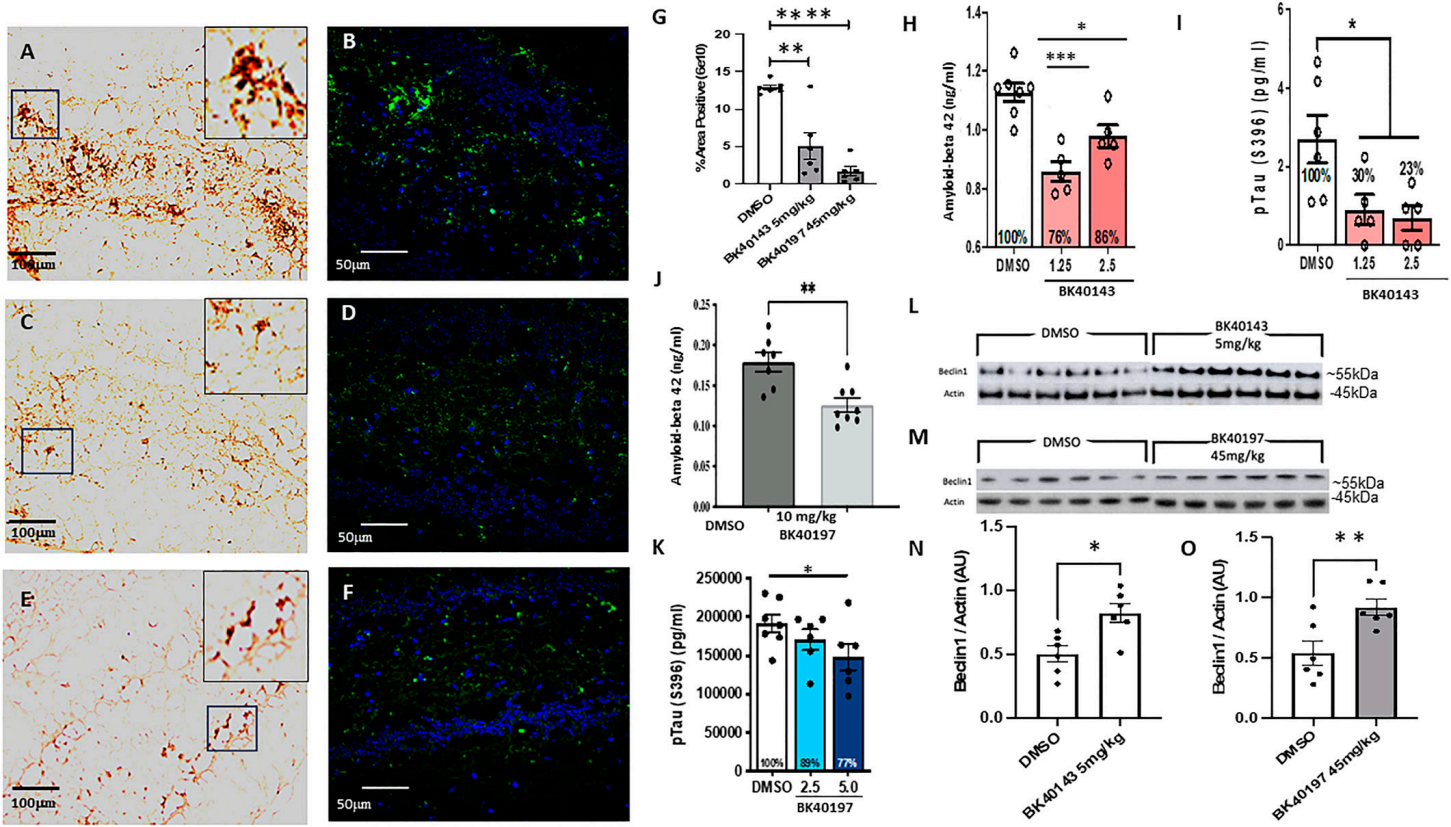
BK40143 and BK40197 reduce amyloid-beta concentrations and induce autophagy in vivo. **(A, B, C, D, E, F, G)** TgAPP mice injected with DMSO displayed significantly greater levels of amyloid-beta (A) 3, 3′ diaminobenzidine staining 20x, (B) AlexaFluor 488 40x compared with mice treated with (C, D) 5 mg/kg BK40143 or (E, F) 45 mg/kg BK40197, quantified in (G) (143: *P* = 0.001, 197: *P* ≤ 0.0001). **(H, I)** ELISA for (H) amyloid-beta in TgAPP mice (1.25 mg/kg: *P* = 0.0001, 2.5 mg/kg: *P* = 0.05) and (I) pTau in Tg4510 mice (1.25 mg/kg: *P* = 0.05, 2.5 mg/kg: *P* = 0.04) treated with BK40143 revealed decreases in protein levels compared with DMSO-treated controls. **(J, K)** ELISA for (J) amyloid-beta in TgAPP mice (*P* = 0.003) and (K) pTau in Tg4510 mice (5.0 mg/kg: *P* = 0.03) treated with BK40197 revealed decreases in protein levels compared with DMSO-treated controls. **(L, M, N, O)** Western blot revealed increased beclin-1 expression in TgAPP mice treated with (L, N) 5 mg/kg BK40143 (*P* = 0.004) and (M, O) 45 mg/kg BK40197 (*P* = 0.05).

To determine the effects of c-KIT inhibition with our compounds on brain amyloid-beta and pTau concentrations in vivo, we first performed immunohistochemistry to visualize amyloid deposition in our treated and untreated animals. We used immunofluorescence and 3, 3′ diaminobenzidine staining using a 6E10 antibody (BioLegend) to observe whether treatment with our compounds had any outcome on amyloid levels. We discovered that treatment with BK40143 (Fig 6C, D, and G) and BK40197 (Fig 6E–G) robustly decreased amyloid concentrations in the dentate gyrus of the hippocampus in treated animals compared with DMSO controls (Fig 6A, B, and G). We further confirmed that TK inhibition reduces concentrations of CNS neurotoxic proteins via ELISA for amyloid-beta and tau using brain lysates taken from both TgAPP and Tg4510 mice. We found that both BK40143 (Fig 6H and I) and BK40197 (Fig 6J and K) treatment significantly reduced Aβ and pTau (396) concentrations compared with DMSO-treated controls. To confirm our screening data that BK40143 and BK40197 bind to various autophagy proteins (mTOR, ULK3, PIKEFYVE, etc.) and induces autophagy in cell-stressed with alpha-synuclein in vitro (Fig 2), we performed protein analysis on brain samples harvested from TgAPP mice. Western blot revealed significant increases in levels of beclin-1, a multi-domain protein integral for the induction of autophagy, in mice treated with BK40143 (Fig 6L and N) and BK40197 (Fig 6M and O) compared with DMSO-treated controls, indicating that drug treatment is sufficient to induce autophagy in vivo, in association with reduced amyloid and pTau. This further identifies c-KIT inhibition as an effective mechanism for clearing neurotoxic protein aggregates in AD brains.

### BK40143 and BK40197 reduce microglial inflammation

**Figure 7.**
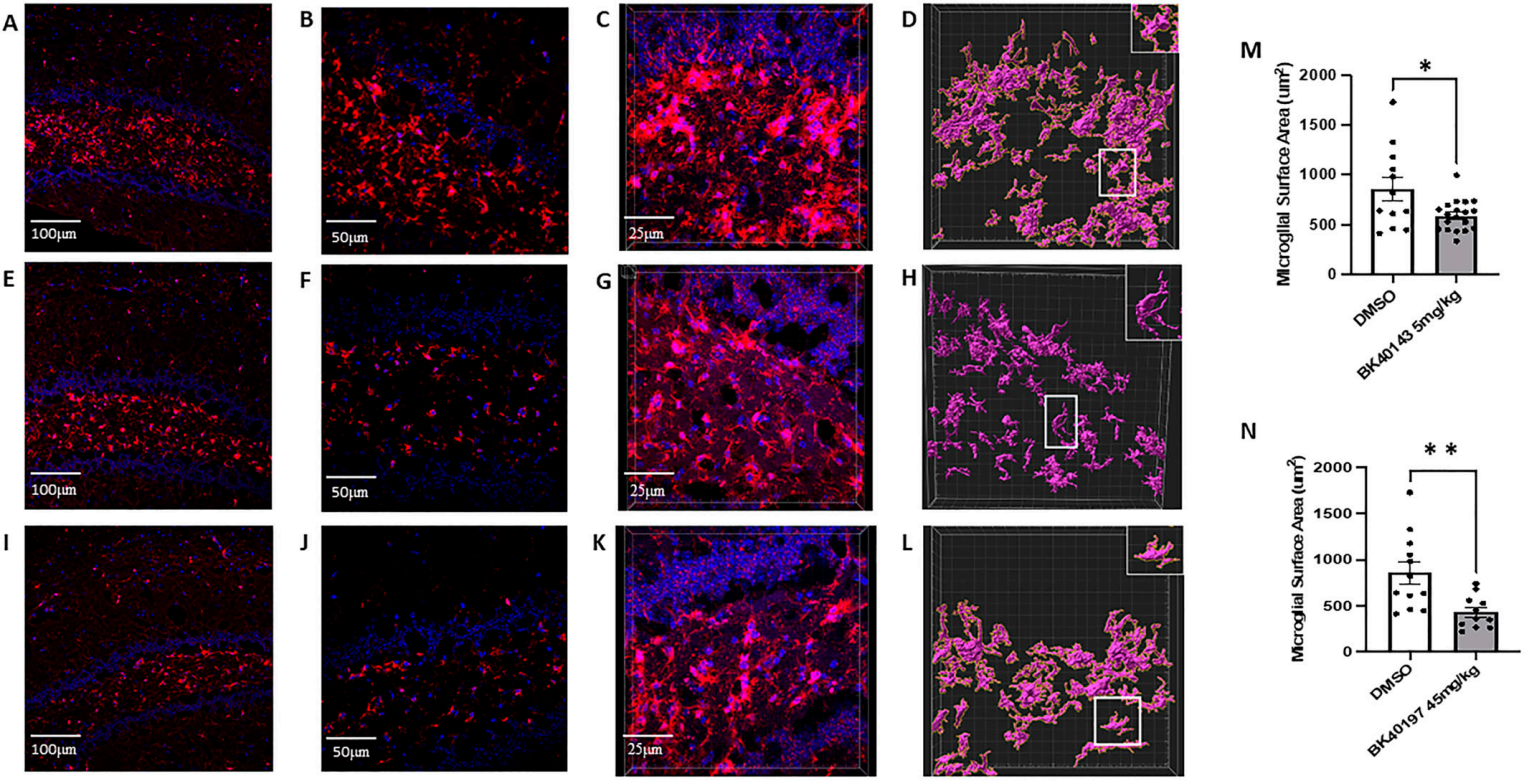
BK40143 and BK40197 alleviate microglial inflammatory morphology. **(A, B, C, E, F, G, I, J, K)** Immunohistochemical staining of IBA1+ microglia revealed increased staining intensity in TgAPP mice treated with DMSO ((A) 20x, (B) 40x, (C) 63x z-stack) compared with mice treated with (E, F, G) 5 mg/kg BK40143 and (I, J, K) 45 mg/kg BK40197. **(D, H, L, M, N)** IMARIS 3D reconstruction and analysis revealed microglia from (D) DMSO-treated mice displayed significantly greater surface area than microglia from mice treated with (H, M) 5 mg/kg BK40143 (*P* = 0.01) and (L, N) 45 mg/kg BK40197 (*P* = 0.004).

To examine the effects of c-KIT inhibitors on microglial activation and subsequent neuroinflammation in vivo, we performed immunohistochemistry and morphological analysis of microglia from the brains of treated and untreated animals. We stained for IBA1, a calcium-binding protein used to identify microglia, before analyzing microglial morphology, a representative feature of activated microglia, using the “surfaces” plug-in in Imaris. We observed a significant reduction in microglial activation in both BK40143 (Fig 7E and F) and BK40197 (Fig 7I and J) treated microglia compared with DMSO controls (Fig 7A and B). We also found significantly reduced surface area of microglia from BK40143 (Fig 7G, H, and M) and BK40197 (Fig 7K, L, and N) treated versus untreated (Fig 7C and D) animals. This identifies c-KIT inhibition as a mechanism for reducing microglial activation and neuroinflammatory phenotypes in AD brains and suggests a multiple mechanism process by which TKIs may be promoting therapeutic benefit.

## Discussion

**Figure S1.**
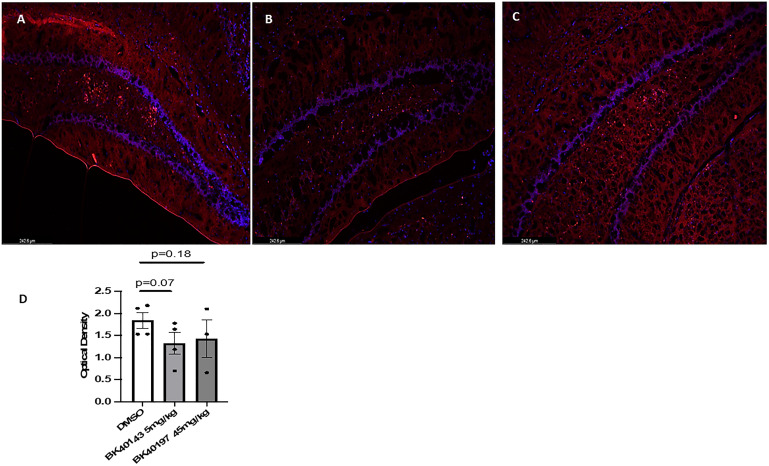
BK40143 and BK40197 reduce microglial PAR2-tryptase in TgAPP mice. **(A, B, C, D)** Proximity ligation assay showing direct PAR2-tryptase interactions in (A, D) DMSO-treated mice compared with (B, D) BK40143-treated and (C, D) BK40197-treated mice compared with. One-tailed unpaired *t* test.

Our data demonstrate a common target for BK40143 and BK40197 that is primarily c-KIT, with considerable variations in BK40197 binding specificity to autophagy-associated non-tyrosine kinases notably PIKFYVE and mTOR. The effects of c-KIT on mast cells have evolved as therapeutic targets for several neurodegenerative diseases. c-KIT signaling is involved in APP phosphorylation and Aβ production (Chen et al, 2019). c-KIT deletion ameliorates AD pathology and masitinib is a c-KIT inhibitor currently under clinical investigation as a potential target in neurodegeneration, neuroinflammation, and cognitive improvement in AD (Folch et al, 2015; Fagiani et al, 2020; Dubois et al, 2023). Masitinib is also in late-stage clinical trials for multiple sclerosis (Arsenault et al, 2022; Vermersch et al, 2022). However, masitinib is a poor brain penetrant and its efficacy may be limited to c-KIT effects on peripheral mast-cell immunity and may not be mediated by multi-kinase inhibition that simultaneously underlies CNS autophagy and neuroinflammation; and there is no evidence that masitinib reduces toxic proteins (Li et al, 2020). Chronic oral treatment with masitinib or genetic depletion of mast cells in APPswe/PSEN1dE9 mice reverse spatial learning performance via recovery of synaptic markers; whereas not reducing amyloid-β or attenuating neuroinflammation (Li et al, 2020), suggesting limited masitinib effects via mast cells inhibition and synaptic protection. A more recent study show that mast cell depletion also ameliorates contextual fear coniditioning in 5xFAD mice and upregulates neuroprotective (via transcriptomic) microglial markers, without impacting other cognitive or anxiety-like behavior or reducing amyloid burden (Lin et al, 2023). Although these studies suggest that mast cells may respond to brain immune signals, masitinib seems to have limited effects. In contrast to masitinib, we show that BK40197 -and previously BK40143-abundantly penetrate the brain and we identify a therapeutic safe dose in animals up to 7.5 and 45 mg/kg for BK40143 and BK40147, respectively. TKs are considered as the central driving force for critical downstream processes leading to mast cell activation (Parravicini et al, 2002; Gomez et al, 2005). Whereas the functional involvement of peripheral mast cells in inflammatory conditions is well established, the role of CNS mast cells is not well understood. Increasing evidence indicate that in the brain, inflammation is strongly involved in the pathogenesis of neurodegenerative and neuropsychiatric disorders (Kempuraj et al, 2017; Traina, 2017; Kempuraj et al, 2019). Mast cells and microglia orchestrate immune responses in AD and PD (Sandhu & Kulka, 2021), suggesting targeting of mast cells as a neuroprotective strategy (Ocak et al, 2019). Microglia and mast cell activation are reported in MPTP-treated models of PD (Selvakumar et al, 2020), whereas increased mast cells were also observed in PD models (Zhang et al, 2021a). In amyotrophic lateral sclerosis (ALS), the inflammatory mechanisms in peripheral motor axon degeneration are attributed to mast cells and neutrophils (Trias et al, 2018). c-KIT inhibition results in reduced pathology in TDP43 models of ALS (Spiller et al, 2018), whereas other studies found that a decrease in c-KIT expression in the spinal cord reduced survival of motor neurons compared with SOD1(G93A) mice, but the amount of motor neurons at end stage is similar (Staats et al, 2015). c-KIT activation leads to atypical mast cell proliferation, maturation, and degranulation, which results in detrimental inflammatory processes such as release of pro-inflammatory factors (Jensen et al, 2008). In both the mouse and human, committed bone marrow mast cell progenitors are released into the bloodstream from where they subsequently migrate into peripheral tissues during which time they mature and become terminally differentiated under the influence of cytokines within the surrounding milieu (Metcalfe et al, 1997). The migration of mast cell progenitors appears to be controlled in a tissue specific manner (Gilfillan & Rivera, 2009) and structural interactions between mast cells and a variety of other cells have been observed (Fig S1A–D). For example, BK40143 and BK40197 reduced mast cell mediators such as tryptase that was shown to be attenuated via proximity ligation assays of microglial protease-activated receptors (PAR2)-tryptase interactions (Fig S1A–D). These responses follow antigen-mediated aggregation of FcεRI on the mast cell surface, a response which can be further enhanced following c-KIT (CD117) activation. Activation of TKs is central to the ability of both FcεRI and c-KIT to transmit downstream signaling events required for the regulation of mast cell activation. Whereas c-KIT possesses inherent TK activity, FcεRI requires the recruitment of Src family tyrosine kinases and Syk to control the early receptor-proximal signaling events (Gilfillan & Rivera, 2009). Src has also been shown to be important for activation of mast cells upon FcεRI aggregation and are co-immunoprecipitated with this receptor (Parravicini et al, 2002). Our novel compounds BK40143 and BK40197 have been shown to engage c-KIT in both the brain and periphery as measured via immunohistochemical staining, Western blot and flow cytometry, identifying c-KIT as a potential peripheral biomarker for c-KIT inhibition. Considering our PK analysis revealed that both BK40143 and BK40197 reach the CNS at adequate levels, the ratio of immature-to-mature mast cells in blood represents maybe a suitable measure for inhibition of c-KIT with our compounds. We demonstrated that treatment with our compounds reduces the ratio of mature mast cells in circulation, indicating engagement of c-KIT. It should be noted, however, that the observed differential effects of BK40143 and BK40197 on c-KIT inhibition and mast cell functioning may be due to insolubility to BK40197 in DMSO, therefore a salt composition of BK40197-HCL was synthesized for future experiments. In addition, mast cells do not exist in circulation at high levels, so future studies examining mast cell maturation in peripheral tissues may further elucidate the effects of c-KIT inhibitors of mast cell proliferation.

Despite their common effect on c-KIT, BK40197 differentially binds to other protein kinases, including PIKFVE and mTOR, that induce autophagic clearance of toxic proteins, in agreement with our animal data showing reduction of amyloid and pTau in TgAPP models and improvement of cognition. PIKFYVE is involved in several neurological diseases and it plays an important role in maintenance of lysosomal functioning, endosomal trafficking, and autophagy, and it was recently shown to be a relevant target to alleviate ALS pathology (Crunkhorn, 2023; Hung et al, 2023). PIKFYVE and PIP5K1A are enzymes targeted as therapies for cancer, as their inhibition has been shown to induce autophagy (O’Connell & Vassilev, 2021; Rong et al, 2012), further suggesting our compounds as inducers of autophagy, as both compounds led to an increase in beclin-1 levels (Sarkar, 2013). mTOR inhibition leads to increase of autophagy flux (Sarkar, 2013), suggesting that treatment with BK40143 may be inducing mTOR-independent autophagy, whereas treatment with BK40197 may be directly stimulating mTOR-dependent autophagy. Furthermore, both BK40143 and BK40197 target ULK3, whose inhibition has been shown to be a major inducer of autophagy (Gonzalez-Rodriguez et al, 2022). Ultimately, investigation into the roles of these kinases in disease pathogenesis may serve to uncover additional mechanisms by which treatment with our compounds improves cognition in TgAPP mice. Collectively, these data suggest that multi-kinase inhibition provides an effective means of alleviating neurodegenerative pathologies (Hebron et al, 2013b, 2014; Mahul-Mellier et al, 2014; Lonskaya et al, 2015; Javidnia et al, 2017; Fowler et al, 2019), although future investigation into the effects of TK and other kinase inhibition may underlie synergistic and/or harmonious mechanisms (autophagy, anti-inflammation, etc.) to clarify how multi-target kinase inhibitors may function to promote therapeutic benefit in certain neurodegenerative diseases.

Using the KinomeScan competitive binding assay, we were able to identify which kinases our drugs targeted from over 450 candidates. Our analysis revealed potential binding to several other kinases believed to be involved with disease pathogenesis: Abl, SYK, and SRC have also been associated with neurodegenerative disease pathology, including microglial activation and tau phosphorylation, and have been investigated as therapeutic targets (Dhawan & Combs, 2012; Hebron et al, 2013b; Mahul-Mellier et al, 2014; Nygaard et al, 2014). Similarly, IRAK1 and LCK are kinases involved in innate and adaptive immunity that regulate numerous inflammatory pathways (Zhang et al, 2023a, 2023b), suggesting that a multi-kinase treatment with BK40143 and BK40197 may exert synergistic multimodal effects to alleviate inflammation in disease models. DDR1 is implicated in fibrotic disorders and DDR1 inhibition has been associated with decreased vascular fibrosis in mouse models of AD (Hebron et al, 2013a, 2014; Fowler et al, 2020; Stevenson et al, 2023), whereas CSF1R is a cell-surface receptor expressed on myeloid cells, including microglia, and has been targeted by therapies aimed at reducing neuroinflammation in neurodegenerative diseases including AD (Olmos-Alonso et al, 2016). Furthermore, MAP4K4 and MEK5 are kinases involved in cell proliferation and oxidative stress and can phosphorylate tau, indicating that treatment with BK40143 and BK40197 may reduce pathological findings at the cellular level via inhibition of these targets (Abe et al, 1997; Jovanovic et al, 2022).

**Figure S2.**
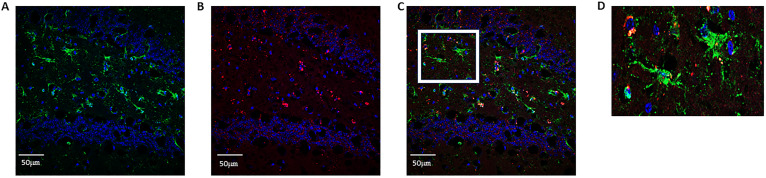
c-KIT is expressed on microglia in Tg APP mice. **(A, B, C, D)** Immunohistochemical staining for (A) IBA1 and (B) c-KIT revealed (C, D) expression of c-KIT on microglia in TgAPP mice.

Whereas our lab has been able to demonstrate increased expression of c-KIT in the hippocampi of AD patients via immunohistochemical staining, the cell subtypes expressing c-KIT in these brains had not been classified. These data are subsequently the first to our knowledge to show that c-KIT is expressed on both neurons and microglia in the TgAPP mouse model of AD, highlighting it as a potential target for alleviating pathology by targeting these cell types. Whereas previous publications have suggested that c-KIT is expressed on microglia during development (Zhang & Fedoroff, 1997; Kierdorf et al, 2013), we were able to show via co-labeling with IBA1 that microglia in the hippocampi of TgAPP mice also express c-KIT, whereas c-KIT signal was also detected in the granule layer of the dentate gyrus (Fig S2A–D). This suggests that c-KIT inhibition with BK40143 and BK40197 may be acting via multiple mechanisms: first, that TKI stimulates autophagic clearance of intraneuronal Aβ and pTau aggregates before their release into the cytoplasmic space, and second, that TKI stimulates microglial autophagy and increased breakdown of phagocytosed Aβ plaques, resulting in improved microglial health and a dampened neuroinflammatory response. Finally, we were able to show that treatment with our compounds results in decreased mast cell proliferation via c-KIT inhibition, identifying a potentially third mechanism by which c-KIT inhibition may be attenuating AD pathology. It has been hypothesized that diseases such as AD that exhibit BBB dysfunction may display increased infiltration of peripheral immune cells, including mast cells (Shaik-Dasthagirisaheb & Conti, 2016). By blocking the development of mast cell progenitors into mature, pro-inflammatory mast cells, we were able to demonstrate via proximity ligation assay that interactions between mast cell tryptase and microglia were decreased, suggesting attenuated mast cell-mediated neuroinflammation. However, further research needs to be performed to examine the origins of these mast cells and whether our drugs are acting on CNS-resident mast cells or infiltrating mast cells from the periphery.

When considering drugs such as our novel TKIs that induce autophagy, the specific pharmacodynamic and pharmacokinetic features need to be carefully evaluated to optimize beneficial drug effects. Autophagy can be thought of as a double-edged sword; we have shown that induction of autophagy is associated with reduced neurotoxic protein burden in TgAPP mice, but stimulating autophagy too vigorously can promote cellular self-cannibalization and cell death, as we see in TKIs used for the treatment of various cancers (Chen et al, 2022; Fu et al, 2022). As such, a delicate balance needs to be struck to promote therapeutic levels of autophagy regarding proteinopathies such as AD. Masitinib has an IC_50_ for c-KIT of at least 200 nM (Dubreuil et al, 2009) and has been shown to promote therapeutic benefit in a phase III clinical trial (Dubois et al, 2023), although whether or not the validated dose is sufficient to induce CNS autophagy is unknown. We have shown that treatment with BK40143 at 5 mg/kg and BK40197 at 45 mg/kg is sufficient to induce autophagic clearance of neurotoxic proteins in vivo. Subsequent investigation is thus warranted to distinguish the exact pharmacodynamic parameters of these drugs to determine the optimal dose for achieving cognitive improvements whereas mitigating potential side effects. TKIs have also been shown to rapidly efflux from the CNS across the BBB at higher doses, suggesting that investigation of our compounds at lower doses in vivo is justified to reveal ideal doses for promoting therapeutic benefit. Plasma binding may also be a factor affecting the efficacy of our compounds, further emphasizing the usefulness of repeating the above experiments at alternative doses to identify optimal doses for treating human AD patients. Lastly, BBB dysfunction is a common feature in AD and may affect the brain penetrance of peripherally administered drugs. Subsequently determined effective doses of therapeutics, including BK40143 and BK40197, should be monitored in AD patients to ensure that altered BBB permeability is not modifying the desired brain concentration of the drug.

## Materials and Methods

### Sex as a biological variable

Sex was not considered as a biological variable, as both male and female mice were used and showed similar results.

### Synthesis of BK40143 and BK40197

We prepared a small group of N-heterocyclic aromatic scaffolds with the goal to improve activity with physicochemical characteristics favorable for brain uptake and crossing of the BBB as we previously reported. Neurotherapeutics with a molecular weight below 500 Da and structures that form less than eight hydrogen bonds with solvating water molecules are generally considered more likely to cross the BBB via lipid-mediated free diffusion. To achieve favorable PK properties for efficient brain uptake, we attempted to offset lipophilic features with a moderate molecular dipole moment and hydrogen bond capacities. All products were prepared in high purity according to ^1^H & ^13^C nuclear magnetic resonance spectroscopy and chromatographic methods.

### Specificity binding kinase screening assay

The KINOME*scan* screening platform (Eurofins DiscoverX Corporation) was performed using active site-directed competition binding assay to quantitatively measure interactions between BK40143 and BK40197 and more than 450 human kinases and disease relevant mutant variants. KINOME*scan* assays do not require adenosine triphosphate and thereby report true thermodynamic interaction affinities, as opposed to IC_50_ values, which can depend on adenosine triphosphate concentration (https://www.eurofinsdiscovery.com/solution/scanmax). BK40143 and BK40197 that bind the kinase active site and directly (sterically) or indirectly (allosterically) prevent kinase binding to the (proprietary Eurofins DiscoverX Corporations) immobilized ligand reduce the amount of kinase captured on a solid support, whereas BK40143 and BK40197 that do not bind the kinase have no effect on the amount of kinase captured. Screening “hits” are identified by measuring the amount of kinase captured in test (BK40143 and BK40197) versus control samples by using a quantitative and ultra-sensitive qRT-PCR method that detects the associated DNA label. BK40143 and BK40197 were screened at 10 µM concentration, and results for primary screen binding interactions were reported as “% Ctrl” (POC), where lower numbers indicate stronger hits and calculated as follows:

$$\left[\frac{test\;compound\;signal-positive\;control\;signal}{negative\;control\;signal-positive\;control\;signal}\right]\times 100$$

*test compound = BK40143 or BK40197*

*negative control = DMSO (100% Ctrl)*

*positive control = control compound (0% Ctrl)*

Based on screening data from of profiled compounds, a proportional relationship between primary screening results and corresponding compound/target affinities can be described as binding constants (Kd values) for the indicated ranges of POC values with tighter binding (higher affinity) interactions associated with lower POC values and weaker binding (lower affinity) associated with higher POC values. Briefly, kinase-tagged T7 phage strains were grown in parallel in 24-well blocks in an *E. coli* host derived from the BL21 strain. *E. coli* were grown to log-phase and infected with T7 phage from a frozen stock (multiplicity of infection = 0.4) and incubated with shaking at 32°C until lysis (90–150 min). The lysates were centrifuged (6,000*g*) and filtered (0.2 μm) to remove cell debris. The remaining kinases were produced in HEK-293 cells and subsequently tagged with DNA for qRT-PCR detection. Streptavidin-coated magnetic beads were treated with biotinylated small molecule ligands for 30 min at RT to generate affinity resins for kinase assays. The liganded beads were blocked with excess biotin and washed with blocking buffer (1% BSA, 0.05% Tween 20, 1 mM DTT; SeaBlock [Pierce]) to remove unbound ligand and to reduce non-specific phage binding. Binding reactions were assembled by combining kinases, liganded affinity beads, and test compounds in 1x binding buffer (20% 0.17x PBS, 0.05% Tween 20, 6 mM DTT; SeaBlock). Test compounds were prepared as 100x stocks in 100% DMSO and directly diluted into the assay. All reactions were performed in polypropylene 384-well plates in a final volume of 0.02 ml. The assay plates were incubated at RT with shaking for 1 h and the affinity beads were washed with wash buffer (1x PBS, 0.05% Tween 20). The beads were then resuspended in elution buffer (1x PBS, 0.05% Tween 20, 0.5 μM non-biotinylated affinity ligand) and incubated at RT with shaking for 30 min. The kinase concentration in the eluates was measured by qRT-PCR.

### In vitro autophagy assay

Human SH-SY5Y neuroblastoma cells were grown and cultured in F12 media with 10% fetal bovine serum, 1% L-glutamine, and 1% pen/strep at normal atmospheric conditions (37°C, 21% O_2_, 5% CO_2_). Upon reaching 50–70% confluence, cells were transduced with a lentivirus containing an alpha-synuclein plasmid and allowed to grow for 8 h. Cells were then treated with an ascending dose (100 nM–10 μM) of BK40143 or BK40197, or DMSO or rapamycin/chloroquine overnight before being harvested and treated with the Abcam Autophagy Assay kit (cat. #ab139484) according to manufacturer’s protocols. Cells were then resuspended in cell staining buffer before being analyzed on a Becton Dickinson LSRFortessas flow cytometer.

### In vivo mast cell analysis

Whole blood samples were harvested from the right ventricle of treated and untreated mice and added to 1,000 U/ml heparin-coated tubes to prevent coagulation. 100 μl of blood was then treated with three flow-conjugated antibodies: 647 anti-mouse CD45 (cat. #103123; BioLegend), PE anti-mouse CD117 (c-KIT) (cat. # 105807; BioLegend), and APC/fire 750 anti-mouse FcεR1α (cat. #134339; BioLegend) for 30 min, then treated with 1x RBC lysis buffer (cat. #420301; BioLegend) for 20 min. Cells were washed and resuspended in cell staining buffer before being analyzed on a Becton Dickinson LSRFortessas flow cytometer. Immature-to-mature mast cell ratio was calculated by dividing the percentage of single-labelled (CD117+) mast cells by the total number of c-KIT expressing cells (CD117+ plus CD117+ FcεR1+). All animal work was approved by Georgetown University Animal Care and Use Committee (GUACUC).

### Pharmacokinetic analysis

The PK profile of BK40197 was investigated after single IP doses of 20 and 45 mg/kg BK40197 to male and female 5–6 mo old C57BL/6 mice compared with DMSO. A solution at 5 ng/ml isotope labelled reference compounds d8-BK40197 was prepared. The solvent used for internal standard (ISTD) dilution is (200 ml/50 ml) acetonitrile/ethyl acetate (ACN/EtOAc). Brain tissue was homogenized in ice water bath and was prepared using weight accurately on analytical balance with 4 decimals. A volume water (MilliQ) was added by adjustable 100–1,000 μl to a concentration at 100 mg brain tissue/ml water. Brain homogenate (25 μl) and plasma sample (25 μl) that was thawed to RT and gently mixed were pipetted to eppendorf tube with lid and 75 μl ISTD added, mixed on vortex and centrifuged for 5 min at 12, 3 RCF. A volume of 25 μl milli-Q water and 75 μl brain or plasma samples were added into a 200 μl PE vial and mixed on vortex and 10 μl and injected on LC-MS/MS. All animal work was approved by GUACUC.

### Drug tolerability assessment

3–4-mo-old WT mice (male and female, C57BL/6) were IP injected with starting doses of 10 mg/kg of BK40143 or BK40197 daily for 5 d before a 2-d washout period. BK40143 dosage was reduced to from 10 to 7.5 mg/kg doses due to rodent mortality, and this dose was continued in 5 d increments up to 70 d. BK40197 dosage was increased in 10 mg/kg increments for 5 d each until the maximum tolerable dose of 50 mg/kg was reached. All animal work was approved by GUACUC.

### Mice

Mice used for experiments and analysis were primarily 12–14-mo-old TgAPP mice expressing the neuronally derived human APP gene, 770 isoform, containing the Swedish, Dutch, and Iowa familial Tg(SwDI) mutations driven by a Thy1 promoter. A mixture of males and females were used for each experiment, as sex was not considered as a biological variable for these experiments. Cohorts consisted of 10 mice per treatment group, and 2–3 cohorts for each drug were used. To investigate the effects of BK40143 and BK40197 on levels of amyloid-beta, we used an additional cohort of 4-mo-old TgAPP mice. To investigate drug effects on levels of phospho-Tau, we used cohorts of 3-mo-old Tg4510 tauopathy mice that express the human P301L tau mutation driven by a CAMKIIα promoter. For behavioral analysis, we also treated human α-synuclein mutant (A53T) mice possessing a mutation to the *SNCA* gene driven by a prion (PrP) promoter to overexpress α-synuclein, of which equal numbers of male and female mice were used. All animal work was approved by GUACUC.

### Drug treatment

BK40143 and BK40197 were kept in powder form at -20°C until ready for use. Drugs were dissolved in DMSO at 1.25, 2.5, or 5 mg/ml for BK40143 and 2.5, 5, 10, or 45 mg/ml for BK40197. Mice were then weighed, and the corresponding volume was IP injected daily within 2 h of the previous day’s injection for 6 wk in the treated mice.

### Novel object recognition

Novel object recognition was carried out using a 50 × 50 cm arena with clear walls covered by brown construction paper. Mice were habituated to the room for 30 min each day, before being allowed to freely explore the arena for 5 min two times, for 3 d. On the 4th d, two identical objects were placed in the arena in opposite adjacent corners, 10 cm from the corner of the arena. Mice were placed along the wall opposite from the objects and allowed to freely explore for 10 min. Approach behavior (extending nose toward the object) toward both objects was recorded manually using handheld stop watches and compared with ensure there was no preference for either neutral object. For the probe trial, one object was removed and replaced with a novel object, and mice were again allowed to freely explore for 10 min, with approach time towards the novel and familiar objects recorded. Trials in which the mice explored for less than 1 s were excluded. Discrimination index was calculated by dividing time spent exploring the novel object by total time exploring both objects and was compared for treated versus control animals.

### Morris water maze

The Morris water maze was performed using a water bath 6 ft in diameter and filled with roughly 1 ft of water. The maze was divided into four quadrants, each with a visual stimulus positioned on the wall of the maze in the center of the quadrant arc. A clear platform was placed in one quadrant just beneath the surface of the water. Mice were then placed in a random non-platform quadrant and allowed to swim freely for 60 s (time was stopped and recorded if the mouse found the platform) before being placed on the platform for 15 s and returned to their cage. This was repeated for each of the three quadrants each day for 4 d. On the 5th d, the platform was removed and mice were placed in the quadrant opposite of where the platform had been and allowed to swim freely for 1 min before being returned to their cage. Time spent in the correct quadrant, latency to the platform, number of platform entries, and number of quadrant entries were recorded.

### Elevated plus maze

The elevated plus maze was performed using a standard set up, with two enclosed arms and two open arms. Mice were placed at the intersection of the open and closed arms and allowed to freely explore for 5 min, two times. Time spent in the open and closed arms and number of entries into the open and closed arms were recorded.

### Nesting

Mice were singly housed in standard cages overnight (6 pm–6 am) with a 3-gram cloth nestlet and allowed to freely behave. The next day, nestlets were weighed and photographed, and amount of nestlet shredding was recorded by three observers based on the following scale: 0—nestlet untouched, 1—nestlet hardly touched (more than 90% intact), 2—nestlet partially torn (50–90% intact), 3—mostly shredded but no identifiable nest, less than 50% intact but less than 90% in a single quarter of the cage, 4—An identifiable but flat nest, more than 90% torn and material is gathered in a single quarter of the cage, 5—A near perfect nest, more than 90% of the nestlet is torn and the nest is a crater, with walls higher than the mouse body height.

### Immunohistochemistry

Staining for amyloid-beta and microglia was performed on left brain hemispheres collected from treated and untreated mice. Brains were cryoprotected with 30% sucrose and sectioned at 30 μm on a cryostat. Sections were then treated with an anti-amyloid beta (6E10, cat. #803001; BioLegend) or anti-IBA1 (cat. # 018-28523; Wako Chemicals) primary followed by either an AlexaFluor secondary (Thermo Fisher Scientific) and DAPI mounting medium (Vector Labs) or a biotinylated secondary and 3, 3′ diaminobenzidine chromogen substrate. Slides were imaged on either a Leica DMi8 light microscope or a Zeiss LSM 800 confocal microscope.

### Western blot

Brain hemispheres from treated and untreated animals were lysed in 1x STEN buffer and protein concentrations were measured via BCA assay. Samples were normalized to 1 μg/μl and added to 2x Laemmli loading buffer + β-mercaptoethanol and boiled for 10 min to denature the protein. 15 μg of sample were then loaded in 4–12% Bis–Tris gels with MES buffer and electrophoresed, before transfer to a nitrocellulose membrane, where they were blocked with 5% dry milk in 1x TBST and incubated overnight with an anti-Beclin-1 primary antibody (cat. #3739S; Cell Signaling) or a phospho-c-KIT antibody (cat. #44-496G; Invitrogen). The next day, the membrane was washed and treated for 1 h with a goat anti-rabbit HRP-linked secondary followed by SuperSignal West Dura Extended Duration Substrate (cat. #37071; Thermo Fisher Scientific) and imaged on an Amersham Imager 600. This procedure was repeated the next day with an anti-mouse actin primary as a loading control.

### ELISA

All samples were analyzed in parallel using the same reagents. A total of 25 μl soluble protein was incubated overnight at 4°C with 25 μl of a mixed bead solution. After washing, samples were incubated with 25 μl detection antibody solution for 1.5 h at RT. Streptavidin–phycoerythrin (25 μl) was added to each well containing the 25 μl of detection antibody solution. Samples were then washed and suspended in 100 μl of sheath fluid. Samples were then run on MAGPIX with Xponent software. The median fluorescent intensity data were analyzed using a five-parameter logistic or spline curve-fitting method for calculating analyte concentrations in samples. Specific pTau ser396 (KHB7031; Invitrogen), aggregated Aβ (KHB3491; Thermo Fisher Scientific), and Aβ42 (KHB3442; Invitrogen) were performed according to manufacturer’s protocol on tissue soluble extracts from brain lysates in 1XSTEN buffer.

### Microglial morphological analysis

63x z-stack images (0.19 μm intervals) of IBA1 stained microglia from treated and untreated mice were collected on a Zeiss LSM confocal microscope. 3D images were then analyzed using the “surfaces” plug-in in Imaris 10.0 to ascertain cell-surface area. Individual microglial surface areas were calculated in the advanced statistics tab of Imaris.

### Proximity ligation assays were performed using the Millipore Duolink PLA protocol

Slides containing two consecutive brain slices were blocked in Duolink blocking solution for 1 h in a slide chamber containing water at 37°C. Blocking solution was then removed and PAR2 (1:250) and tryptase (1:250) primary antibodies were added at the concentrations specified above and incubated at 4°C overnight. The next day, primaries were removed and slides were washed twice for 5 min in wash buffer before addition of plus and minus probes for incubation at 37°C in a humid chamber for 1 h. Slides were then washed again twice for 5 min in wash buffer before addition of ligation buffer for incubation at 37°C in a humid chamber for 30 min. Next, slides were washed twice for 5 min in wash buffer, and polymerase was added for incubation at 37°C in a humid chamber for 100 min. Finally, slides were washed twice for 10 min in wash buffer and mounted using DAPI mounting medium and a glass coverslip. Slides were imaged on a Leica Dmi8 light microscope.

### Statistical analysis

All statistics were calculated in GraphPad Prism 9. Two-tailed unpaired *t* tests were used to compare treatment groups. Ordinary one-way ANOVAs were used to examine the effects of our drugs at different doses in vitro. Two-way ANOVA was used on the Morris water maze to compare group differences over the course of the trials.

### Study approval

All animal studies were approved by the Georgetown Animal Care and Use Committee GUACUC. This is not a clinical trial.

## Supplementary Material

Reviewer comments

## Data Availability

The data supporting the current study are available from the corresponding author on request.
