# Supplementary figures and images for: c-KIT inhibitors reduce pathology and improve behavior in the Tg(SwDI) model of Alzheimer’s disease
Source: Life Sci Alliance. 2024 Jul 15;7(10):e202402625. doi: 10.26508/lsa.202402625 (PMC11249953; doi:10.26508/lsa.202402625)

## Slide 1
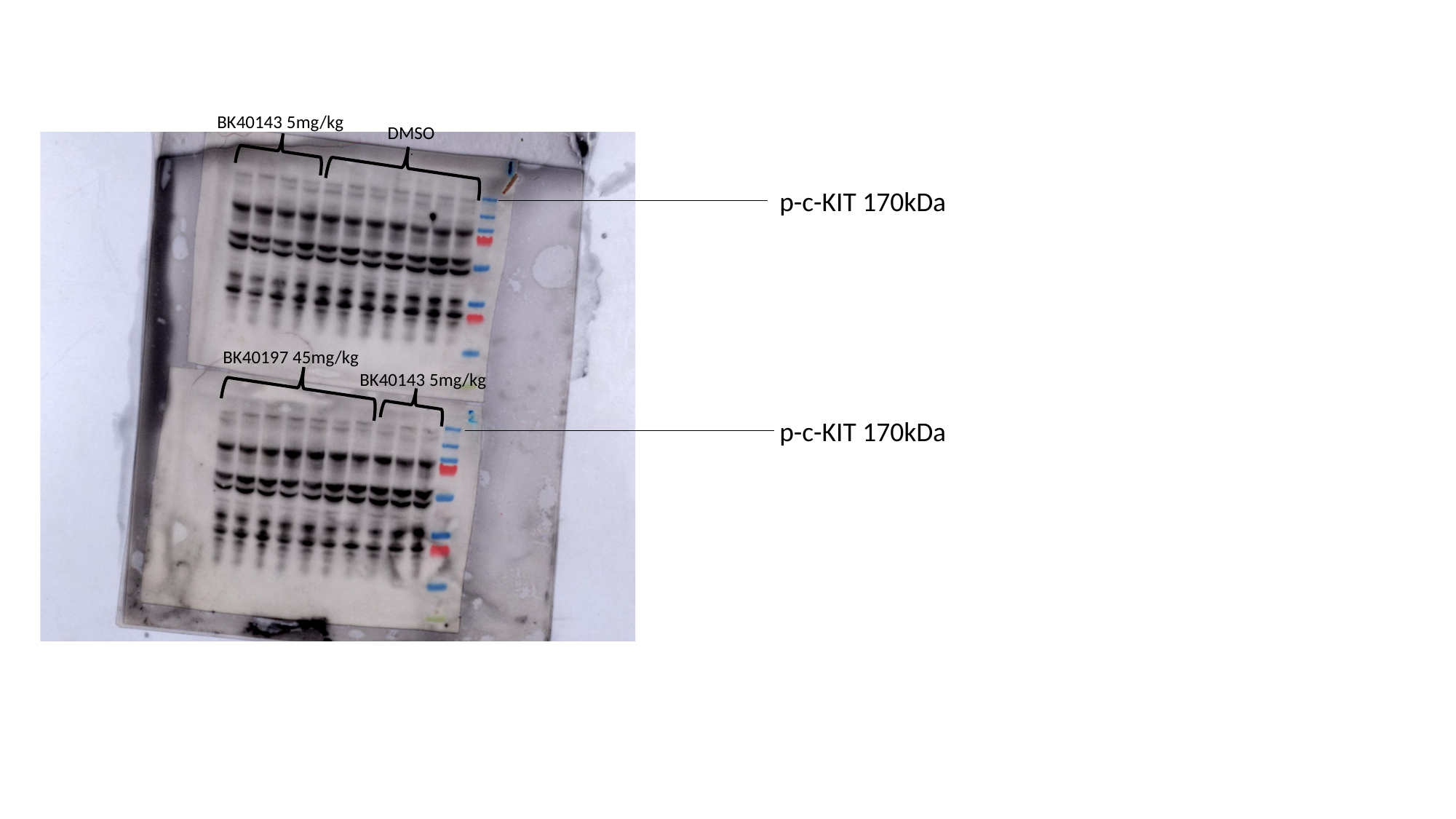

BK40143 5mg/kg
DMSO
p-c-KIT 170kDa
BK40197 45mg/kg
BK40143 5mg/kg
p-c-KIT 170kDa

## Slide 2
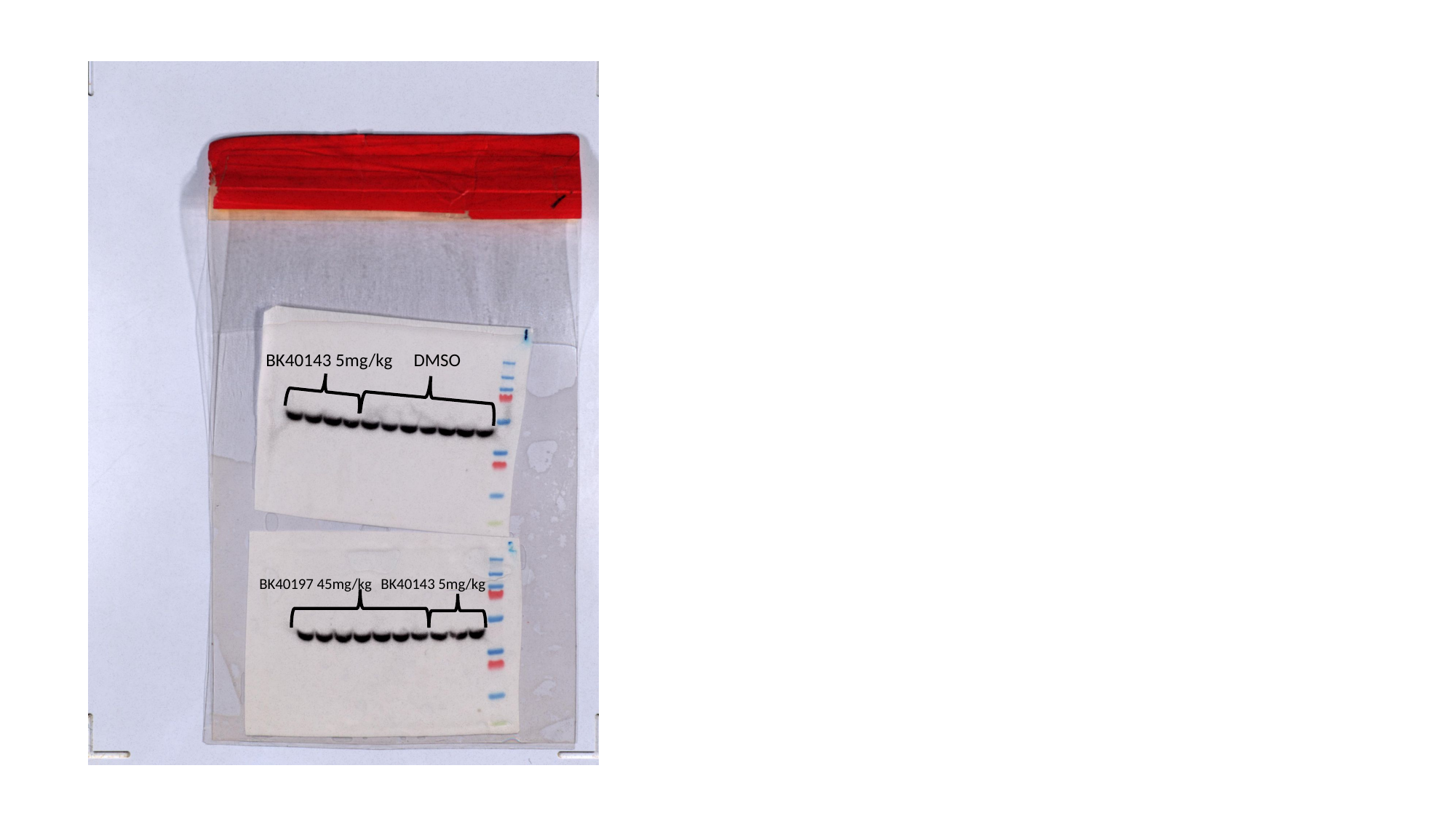

BK40143 5mg/kg
DMSO
BK40197 45mg/kg
BK40143 5mg/kg

Supplement: Supplementary file 3 [file LSA-2024-02625_SdataF3.pptx]
